# Facilitators and barriers to decentralized Long COVID platform clinical trials using repurposed drugs

**DOI:** 10.3389/fpubh.2026.1822376

**Published:** 2026-09-30

**Authors:** Marco Schito, Stacey Coe, Jaime Seltzer, Hannah E. Davis, Vidula Sukhatme, Theodore W. Johnson II, Judy Stone, Alexander D. Truong, Linda N. Geng, Tiffany A. Walker

**Affiliations:** 1 CURE Drug Repurposing Collaboratory, Critical Path Institute, Tucson, AZ, United States; 2 Myalgic Encephalomyelitis Action Network, Santa Monica, CA, United States; 3 Stanford University School of Medicine, Stanford, CA, United States; 4 Patient Led Research Collaborative, New York, NY, United States; 5 Global Cures, Inc., Newton, MA, United States; 6 Emory University School of Medicine, Atlanta, GA, United States; 7 Retired M.D., Cumberland, MD, United States

**Keywords:** clinical trial design, decentralized clinical trials, Long COVID, patient reported outcome measures, repurposed drugs

## Abstract

Long COVID is an emerging chronic condition that results in substantial impairments in quality of life, physical function, and ability to maintain gainful employment. There are no FDA-approved treatments for Long COVID, and recent institution-based clinical trials have not yielded effective treatments. Current standard of care involves trial-and-error drug repurposing that warrants evaluation in rigorous clinical trials. Here we describe potential advantages of decentralized repurposing drug trials as an alternative or complementary approach to institution-based trials. Additionally, we describe lessons learned when launching a local feasibility pilot trial that may inform the design of future decentralized trials in the Long COVID field.

## Introduction

Long COVID is a chronic condition estimated to affect 400 million individuals globally, with over 60% experiencing long-term functional impairments and most unable to return to work at full capacity (1–4). Long COVID symptoms of fatigue, post-exertional malaise, dysautonomia, and brain fog can limit travel to clinical visits and enrollment in research studies, therefore decentralized clinical trials are a promising alternative to traditional study designs. As there are no FDA-approved therapies for Long COVID, many providers turn to symptom-based treatment using off-label drugs. Some treatments, such as low dose naltrexone, are not readily available, requiring use of compounding pharmacies to supply appropriate dosages. Off-label treatments warrant testing in rigorously designed clinical trials to guide clinicians on the right therapies to use and for whom, and this pathway holds promise for improving accessibility of treatments with limited commercial availability.

Although several underlying pathobiological mechanisms have been discovered, it remains unclear which processes are driving Long COVID and how these mechanisms correlate with distinct phenotypes (5, 6). This complexity and etiologic uncertainty impedes drug development and makes drug repurposing and platform clinical trials more attractive, wherein multiple previously-approved drugs for other indications can be trialed in parallel against a single control. Given the burden and high prevalence of Long COVID it is clear that effective therapies are urgently needed, and decentralized drug repurposing clinical trials offer distinct advantages.

To this end, we designed a patient-centered decentralized adaptive platform randomized placebo-controlled trial investigating repurposed drugs currently being prescribed in Long COVID specialty care where we assessed global function and symptom-specific outcomes through patient-reported outcome measures (PROs). Here we discuss the advantages of this approach and the barriers encountered when launching a local pilot assessing feasibility and acceptability of scaling a decentralized trial [NCT05946551].

## Decentralization

Although remote clinical trials, conducted entirely via telemedicine, are a relatively novel approach to identifying effective drug therapies, several similar studies have demonstrated strengths of the decentralized approach (7), including ACTIV-6, a platform trial evaluating multiple repurposed drugs in the treatment of COVID-19. This trial demonstrated the feasibility of rapid participant enrollment in low-touch trials investigating repurposed medications. Although results of all treatments were null, this provided timely guidance on drugs that were gaining popularity in clinical practice, particularly Ivermectin (8). Traditional trials are predominately limited to recruitment of subjects who seek care at large academic institutions. Remote studies may facilitate rapid enrollment as recruitment is not dependent on proximity to study sites, promoting recruitment of marginalized populations with limited healthcare access as well as those with debilitating Long COVID symptoms which limit travel to study sites. This representative recruitment facilitates inclusion of populations that are typically underrepresented in institution-based trials, such as rural and low-income urban populations and ensures the greatest impact for those affected by this disease.

Decentralized trials promote cost-effectiveness by limiting use of facility resources and overhead expenditures, and they minimize participant burden with flexible data collection windows. Furthermore, these pragmatic approaches offer opportunity to leverage real world evidence (RWE) and infrastructure, including health tracking data from mobile devices and outcomes measures from electronic health records (EHR). Although health data standardization is limited across healthcare systems, this may improve over time with expanded use of common data models, facilitating RWE applications in the future.

We identified several barriers when launching our feasibility trial, including inability to enroll across state lines. State medical licensing prevented site principal investigators (PIs) from extending enrollment to other states due to prescribing restrictions, negating some benefit of virtual operations. Similarly, our institution’s investigational drug pharmacies (IDPs) were unable to ship investigational products (IPs) across state lines. Additionally, we encountered challenges with marketing to populations external to our institution. Despite these challenges, nearly half of participants enrolled in our pilot feasibility study resided in rural towns and virtual operations allowed for recruitment of severe Long COVID cases that would have otherwise been unable to participate in in-person visits. In an exit survey, all participants reported preference for virtual participation over in-person attendance at an academic center. All reported that they were satisfied with opportunities to interact with study staff and report adverse events, and none reported challenges with online surveys or medication administration.

Although this trial was designed to investigate repurposed drugs with favorable safety profiles conducive to limited clinical monitoring, we recognized that monitoring of serious adverse events (SAE) was limited to patient reporting as we have limited access to healthcare records outside of our institution, particularly for rural patients or those whose electronic medical records did not integrate fully with ours. Although this can be true in traditional trials that recruit directly from an academic institution given prevalent fractured care, this challenge is likely amplified in virtual trials with broad geographical recruitment.

Additionally, lack of in-person study visits limits measurement of biomarkers to explore impact of repurposed drugs on pathobiological drivers of disease remission or persistence. Incorporation of nationwide laboratories was considered in the study design, but it was decided that this would negate the benefits of virtual operations to the patient and would be logistically challenging for nationwide implementation. However, there remain no universal biomarkers for Long COVID. This limitation should be readdressed as validated biomarkers for Long COVID become available.

## Repurposed drugs

Prior FDA approval of the drugs being studied facilitates direct deployment of Phase II/III clinical trials using affordable treatments with well-tolerated safety profiles that can be rapidly integrated into Long COVID standard of care, potentially reducing drug development time, resources, and costs. Despite these potential benefits, the repurposed drug field has encountered barriers due to lack of funding opportunities. This stems, in part, from limited financial incentives for manufacturers to engage in trials of off-patent drugs and an emphasis on new drug development from scientific funding agencies, resulting in sponsor/investigator-led trials with limited resources for trial implementation and marketing authorization procedures (9). We selected two drug classes, naltrexone and histamine receptor antagonists, that were currently used off-label in Long COVID clinical practice.

### Low-dose naltrexone

Naltrexone is a safe synthetic opioid antagonist that may have immune modulatory effects at low doses (1.5–4.5 mg/day) (10). Low-dose naltrexone (LDN) has gained popularity in Long COVID clinics, with nearly 14% of Long COVID patients receiving an LDN prescription and over half self-reporting improvement in ≥1 Long COVID symptom (11). As target dosages deviate from FDA-approved dosing and current manufacturing practices and may pose potential safety concerns, our trial required LDN compounding and investigational new drug (IND) review, including chemistry, manufacturing, and controls (CMC) monitoring and certificate of analysis (CoA). This required contracting with an external pharmacy capable of microdose compounding and experience with adherence to regulatory standards. The additional regulatory monitoring partially negated the cost-effectiveness anticipated from drug repurposing, and due to logistical and budgetary constraints, we were unable to proceed with LDN in the pilot feasibility trial.

### Histamine receptor antagonists

Histamine receptor antagonists (HRA), such as cetirizine and famotidine, have garnered interest as possible drug candidates for targeting unmitigated mast cell activation and regulation of cellular inflammatory mediators that may contribute to Long COVID symptomatology, and have shown benefit for an array of Long COVID symptoms (12, 13). Our pilot study encountered barriers achieving enrollment goals as many potential candidates were already prescribed HRAs for Long COVID. These medications are low-risk, affordable, and broadly available without prescription, so there was little incentive for individuals to risk randomization to placebo arm if over-the-counter use may provide relief. Anecdotally, all enrollees reported wanting to advance Long COVID care as their reason for opting in to the study rather than being motivated by receiving a medication prescription.

## Patient-reported outcome measures

As no definitive biomarkers have been identified and validated to monitor response to treatments in Long COVID clinical trials and remote data collection was required for the decentralized design, we proposed patient-reported outcome (PRO) measures as primary and secondary endpoints, incorporating input from individuals with lived experience. Accounting for symptom heterogeneity in Long COVID, we initially opted to evaluate change in global function using the WHODAS 2.0 as the primary endpoint under the guidance of patient advisors. However, one caveat of this approach is it assumes that investigational product (IP) pharmacology will equally impact the mechanisms driving individual symptoms. If this is not the case, it is possible that some symptoms may improve, some may remain unchanged, and some may potentially worsen, which may result in a null study that obscures symptoms-specific treatment effect size. Additionally, regulatory agencies raised concerns with the specificity of global measures, pointing out that this outcome is too vague and does not clarify what aspect of the disease improves outcomes. As our understanding of Long COVID evolves, it appears that enrollment by symptom or phenotype (i.e., cognitive impairment, ME/CFS, POTS, etc.) with corresponding symptom-specific PRO primary endpoints may provide greater specificity. However, further investigation is needed in this area to develop and validate PROs specific to Long COVID manifestations.

## Discussion

Decentralized platform repurposed drug trials offer a promising complement to ongoing institution-based Long COVID clinical trials. Despite the many benefits of decentralized trials there are clear lessons learned from our pilot feasibility study and limitations of this methodology that can inform the design of Long COVID clinical trials currently under development to promote successful implementation and reduce bias (Table 1). Novel approaches to recruitment may be necessary to ensure rural and urban populations are aware of trial opportunities. Large-scale campaigns that engage patient advocacy groups and leverage social media and advertisements may improve enrollment. Although we did not encounter issues with fraudulent enrollment, this is a potential vulnerability of decentralized trials and rigorous procedures should be instituted to ensure identity verification, which may be achieved through two-factor identification. Similarly, telemedicine can be leveraged to surveil IP accountability measures to ensure study outcomes are reflective of treatment effects to reduce bias.

**Table 1:** Facilitators and barriers to decentralized repurposing drug trial implementation.

Characteristics	Facilitators	Barriers	Recommendations
Decentralized trial design			
Recruitment and enrollment	Remote design promotes inclusion of marginalized and underrepresented populations	Barriers to enrollment across state lines thwarted expanded access opportunities Challenges with external recruitment strategies limited enrollment	Improve reciprocal state medical licensing agreement Engage patient advocacy groups and leverage social media for large scale recruitment campaigns
Infrastructure	Reduced staff and facility resources requirements Affordable, repurposed IP Reduced drug research and development (R&D)	Pharmacy regulatory and shipping requirements warranted additional infrastructure	Contract with external pharmacies capable of adherence with regulatory standards and shipping across state lines
RWE application	Can leverage RWE to improve generalizability and reduce costs	Limited data standardization	Can improve data harmonization through common data models
Trial monitoring	Limited monitoring required for patient-entered data Low-touch procedures permissible for trials with safe, previously FDA approved IPs	Monitoring of SAE was limited to patient reporting due to limited access to external EHR Remote operations may create opportunities for fraudulent enrollment and limited supervision of IP	Consider dual factor authentication to limit fraudulent enrollment Leverage telemedicine to institute rigorous IP accountability measures
Regulatory compliance	Prior FDA approval facilitates direct deployment and reduced R&D	Compounded drugs require IND approval through acquisition of CMC and COA data	Investigators should account for additional regulatory support needed in off-patent repurposed drug trials
Repurposed drugs			
Low Dose Naltrexone	Affordable, favorable safety profile, may have immune modulatory effects New disease indication may assist with insurance coverage and promote interest in manufacturing pertinent dosages	Compounded drugs require IND process with CMC and COA Additional regulatory monitoring partially negated the cost-effectiveness anticipated from drug repurposing	Contract with an external pharmacy capable of adherence to regulatory standards
Histamine Receptor Agonists	Affordable, favorable safety profile, regulates cellular inflammatory mediators	Boad availability limited participant interest	Consider that real-world practice patterns and readily available medications may impact recruitment goals
Outcome measures			
Patient-reported outcome (PRO) measures	Addresses outcomes of interest to patient community	No validated tools in Long COVID Symptom and disease heterogeneity may impact sensitivity and specificity Regulatory agencies raised concerns about ambiguity of global measures	Further investigation is needed to develop and validate PRO measures
Biomarkers	Provides objective endpoints to assess efficacy and drive treatment recommendations	No validated or universally accepted biomarkers in Long COVID Requires significant infrastructure for decentralized collection and processing that will impact cost effectiveness	Further investigation is needed to develop and validate surrogate biomarkers Universal laboratory infrastructure will need to accommodate participants in rural and resource-limited environments

Decentralized trials would have greater impact if reciprocal state medical licensing agreements continue to expand, allowing clinicians licensed in one state to prescribe IP to residents of other states that offer mutual licensing. In addition, commercial investigational drug pharmacies will need to be used if IP is shipped across state lines. As CMC and CoA regulations will be required for trials incorporating compounded drugs, it would be best to contract with a pharmacy that can serve in both capacities, and the costs need to be accounted for during trial development. If biomarker development or monitoring is incorporated as secondary or exploratory endpoints, universal laboratory infrastructure will need to be coordinated that accommodates participants in rural and resource-limited areas to ensure equitable access to trial enrollment. It may be necessary to consider home-based collection of biospecimens, so that participants who are house bound, may participate. Sponsor/investigators seeking funding should account for additional regulatory support needed in off-patent repurposed drug trials and be aware that trials previously approved drugs may be required to be conducted under an IND.

When selecting treatments for trials, investigators should consider that real-world practice patterns and readily available medications may impact recruitment goals. Although, investigating these drugs is still of paramount importance to clarify phenotype-based efficacy and establish new disease indications, which may assist with insurance coverage and promote interest in manufacturing new dosages for treatments, such as low dose naltrexone. Pragmatic trials incorporating RWE should employ common data models to optimize data harmonization. Finally, until biomarkers are developed, PROs serve as the mainstay of primary endpoints in Long COVID. There is improved specificity by evaluating symptom-based PROs that correspond with participants’ most burdensome symptom/phenotype; however, there should be continued efforts on development of these measures to ensure validity, reliability, and reduce bias.

Over 5 years following the COVID-19 pandemic, there remain no proven effective treatments for Long COVID, underscoring the urgency to expand clinical trials efforts. Decentralized clinical trials offer an excellent complement to site-based clinical trials, addressing many of the challenges encountered in traditional trials. These lessons learned can be incorporated in design and planning phases to improve efficiency and optimize successful implementation.

## Data Availability

The original contributions presented in the study are included in the article/supplementary material, further inquiries can be directed to the corresponding author.
